# Exploring the Potential of Montmorillonite as an Antiproliferative Nanoagent against MDA-MB-231 and MCF-7 Human Breast Cancer Cells

**DOI:** 10.3390/cells13020200

**Published:** 2024-01-22

**Authors:** Alireza Ghannad Sabzevari, Hossein Sabahi, Mohsen Nikbakht, Mehdi Azizi, Hassan Dianat-Moghadam, Zohreh Amoozgar

**Affiliations:** 1 Department of Tissue Engineering and Biomaterials, Faculty of Advanced Medical Sciences and Technologies, Hamadan University of Medical Sciences, Hamadan 6517838736, Iran; ghannad@umsha.ac.ir (A.G.S.); mehdi.azizi6815@gmail.com (M.A.); 2 Department of Life Science Engineering, Faculty of New Sciences and Technologies, University of Tehran, Tehran 1439957131, Iran; hsabahi@ut.ac.ir; 3 Hematology Oncology and Stem Cell Transplantation Research Center, Tehran University of Medical Sciences, Tehran 1411713135, Iran; 4 Department of Genetics and Molecular Biology, School of Medicine, Isfahan University of Medical Sciences, Isfahan 8174673461, Iran; dianat.h@med.mui.ac.ir; 5 Pediatric Inherited Diseases Research Center, Isfahan University of Medical Sciences, Isfahan 8174673461, Iran; 6 Edwin L. Steele Laboratories for Tumor Biology, Department of Radiation Oncology, Massachusetts General Hospital and Harvard Medical School, Boston, MA 02114, USA

**Keywords:** montmorillonite, antiproliferative effect, MDA-MB-231, breast cancer, anticancer nanoparticles

## Abstract

Unlike MCF-7 cells, MDA-MB-231 cells are unresponsive to hormone therapy and often show resistance to chemotherapy and radiotherapy. Here, the antiproliferative effect of biocompatible montmorillonite (Mt) nanosheets on MDA-MB-231 and MCF-7 human breast cancer cells was evaluated by MTT assay, flow cytometry, and qRT-PCR. The results showed that the Mt IC_50_ for MDA-MB-231 and MCF-7 cells in a fetal bovine serum (FBS)-free medium was ~50 and ~200 µg/mL, and in 10% FBS medium ~400 and ~2000 µg/mL, respectively. Mt caused apoptosis in both cells by regulating related genes including *Cas-3*, *P53*, and *P62* in MDA-MB-231 cells and *Bcl-2*, *Cas-8*, *Cas-9*, *P53*, and *P62* in MCF-7 cells. Also, Mt arrested MCF-7 cells in the G0/G1 phase by altering *Cyclin-D1* and *P21* expression, and caused sub-G1 arrest and necrosis in both cells, possibly through damaging the mitochondria. However, fewer gene expression changes and more sub-G1 arrest and necrosis were observed in MDA-MB-231 cells, confirming the higher vulnerability of MDA-MB-231 cells to Mt. Furthermore, MDA-MB-231 cells appeared to be much more vulnerable to Mt compared to other cell types, including normal lung fibroblast (MRC-5), colon cancer (HT-29), and liver cancer (HepG2) cells. The higher vulnerability of MDA-MB-231 cells to Mt was inferred to be due to their higher proliferation rate. Notably, Mt cytotoxicity was highly dependent on both the Mt concentration and serum level, which favors Mt for the local treatment of MDA-MB-231 cells. Based on these results, Mt can be considered as an antiproliferative nanoagent against MDA-MB-231 cells and may be useful in the development of local nanoparticle-based therapies.

## 1. Introduction

Despite advances in diagnosis and treatment, breast cancer is still the leading cause of cancer-related death in women [1,2], highlighting the importance of developing new therapeutic strategies for this disease.

MDA-MB-231 and MCF-7, two well-characterized human breast cancer cell lines, differ from each other in terms of proliferation rate, metastasis rate, and their response to therapy. MCF-7 cells are non-metastatic and responsive to hormone therapy. In contrast, MDA-MB-231 cells, with a higher proliferation rate, are metastatic and unresponsive to hormone therapy and often show resistance to chemotherapy and radiotherapy [3,4]. Therefore, there is a need for new strategies to fight the highly aggressive MDA-MB-231 breast cancer.

An emerging alternative strategy to treat cancers, which are resistant to current therapies, is the use of nanoparticles acting as antiproliferative agents against cancer cells [5,6,7,8]. To date, various inorganic nanoparticles have been developed for suppressing human breast cancer cells. However, while the majority of these nanoparticles have been shown to be able to suppress MCF-7 cells, there have been few reports on nanoparticles capable of suppressing MDA-MB-231 cells [8,9,10]. In addition to being effectively toxic to MDA-MB-231 cells, nanoparticles should be biocompatible and less toxic to other cells; this was often neglected in previous studies [1,11,12].

Clay minerals have been used in pharmaceutical products for centuries and are emerging as an efficient tool for fighting cancer [13,14]. Recently, clay minerals, such as montmorillonite (Mt), halloysite, laponite, kaolinite and layered double hydroxide, as nanocarriers of imaging/therapeutic agents were successfully injected into the circulation for diagnosis and/or systemic treatment of cancers [13,15,16,17,18,19,20]. In addition, clay minerals as nanocarriers of genes/drugs were successfully injected into tumors or tumor resection sites for local cancer treatment, thereby increasing the efficacy and reducing the systemic toxicity of the therapeutic agents [21,22,23,24,25]. Mt, a phyllosilicate consisting of nanosheets with ~1 nm thickness and ~30 nm to several microns in diameter, is FDA-approved for oral, transdermal, rectal, and vaginal use and has long been applied in pharmaceutical products as an active/excipient ingredient [26]. Baek et al. [27] showed that following the oral administration of 1000 mg/kg of Mt in mice, the Mt concentration reached 26 μg/mL in the plasma and Mt was subsequently eliminated from the circulation, without any accumulation in the critical organs or any acute toxicity in the mice. Most studies using rodents also showed a lack of systemic toxicity, even with an oral dose of 5000 mg/kg [28]. In another study, following 143 mg/kg oral and 14.3 mg/kg intravenous administration of Mt daily for 3 days and then performing histopathological, histochemical, hematological and blood biochemical analyses, Lee et al. [29] concluded that Mt was non-toxic to Wistar rats. Considering the biocompatibility of Mt, recently, many researchers have used Mt as an oral nanocarrier of drugs, including chemotherapeutics [30,31,32,33,34,35]. Also, Mt has been recently used as a nanocarrier of imaging/therapeutic agents in injectable anticancer products. For instance, an injectable intratumoral hydrogel composed of Mt, gemcitabine drug, and biodegradable polymers was prepared for the local treatment of pancreatic tumors [25]. An injectable gel consisting of magnetic Mt, doxorubicin drug, and gelatin was prepared for the postoperative treatment of hepatic tumor resection sites [22]. Intravenous injectable nanocomposites composed of Mt, mitoxantrone drug and FePt nanoparticles were developed to simultaneously image and treat hepatic tumors [15]. Superparamagnetic nanocomposites consisting of Mt, Fe_3_O_4_, and *Kappaphycus alvarezii* extract were prepared for the intravenous delivery of anticancer drugs [36]. Finally, an intravenous injectable nano-radiopharmaceutical was developed using Mt for breast cancer imaging [37].

Recently, we evaluated the potential of Mt as an antiproliferative nanoagent and found that Mt possesses intrinsic antiproliferative activity. It inhibits the proliferation of HepG2 (liver cancer), HT-29 (colon cancer), and MRC-5 (normal lung fibroblast) cells in different ways and varying degrees, as the Mt IC_50_ was increased for HepG2, HT-29, and MRC-5 cells, respectively [38]. In the present study, the antiproliferative activity of Mt in MDA-MB-231 and MCF7 cells was investigated for the first time at the cellular and molecular levels by MTT assay, flow cytometry, and qRT-PCR, and then compared with that in MRC-5, HepG2 and HT-29 cells.

## 2. Materials and Methods

### 2.1. Materials

Bentonite was obtained from Poodrsazan Company, Tehran, Iran. The chemical composition of bentonite is shown in Table S1. Fetal bovine serum (FBS), trypsin-ethylenediaminetetraacetic acid (EDTA) 1×, penicillin, streptomycin, and cell culture media (DMEM and RPMI 1640) were provided by Gibco Company, Grand Island, NY, USA. The other chemicals were supplied by Sigma Company, St. Louis, MO, USA.

### 2.2. Mt Preparation and Characterization

Mt was extracted from bentonite and then purified with NaCl as follows: An aqueous dispersion of bentonite (6 g/L) was sonicated for 20 min at 20 KHz, agitated for 24 h, resonicated, and settled for 48 h. Afterwards, the supernatant was centrifuged in a horizontal rotor for 10 min at 200× *g*. NaCl up to 1 M in concentration was added to the resulting supernatant containing Mt, and it was shaken for 24 h and subsequently centrifuged for 20 min at 23,669× *g.* The Mt pellet was shaken in a 1 M NaCl solution and precipitated two more times in the same manner. Then, the excess ions were removed from the Mt pellet by successively dispersing it in deionized water and precipitating it by centrifugation. After reaching the supernatant conductivity of less than 10 μS/cm, measured using a DZS-708 Multi-parameter Analyser (Inesa, China), the Mt pellet was freeze-dried.

Crystallographic data were collected using an X’Pert-MPD Philips (Amsterdam, The Netherlands) X-ray diffractometer with Cu Kα radiation. Zeta potential and hydrodynamic particle size were determined using a Malvern (Malvern, UK) Zetasizer Nano ZS. Field emission scanning electron microscope (FESEM) images were taken using a JEOL (Brno, Czech Republic) JSM-6360 Mira-3 instrument. For the microscopic analysis, Mt was dispersed in deionized water (0.8 wt.%), diluted with acetone (250-fold), and then finally air-dried on a glass slide. Cation exchange capacity (CEC) was measured utilizing the Lorenz method [39].

### 2.3. Cell Culture

MDA-MB-231 (NCBI code: C578), MCF-7 (NCBI code: C135), and MRC-5 (NCBI code: C125) cells were obtained from the Pasteur Institute Cell Bank, Tehran, Iran. The cells were cultured in media supplemented with penicillin (100 IU/mL), streptomycin (100 μg/mL), and heat-inactivated FBS (10%), and incubated in a humidified atmosphere containing 5% CO_2_ at 37 °C. Cell doubling time (DT) was determined as described by Stepp et al. [4].

### 2.4. MTT Assay

The cells at a density of 2500 cells/well were seeded in 96-well plates. Following 24 h incubation, the medium was removed, fresh supplemented media containing different concentrations of Mt (12–4000 μg/mL) were added to the wells, and the cells were incubated for 24 or 48 h. Then, 0.1 mL of medium containing 0.5 mg/mL of MTT (3-[4, 5-dimethylthiazol-2-yl]-2, 5 diphenyl tetrazolium bromide) was added to each well. After a 3–4 h incubation period, the medium was removed, and 0.1 mL of DMSO was added to each well. Finally, the absorbance at 570 nm was read using a Bio-Rad (Hercules, CA, USA) microplate reader. To eliminate the possible effect of Mt size and enhance the accuracy of cell experiments, one Mt preparation was used to make a stock of well-dispersed Mt suspension (10,000 µg/mL in the medium). The stock was then applied to prepare the different concentrations of Mt (12–4000 μg/mL in the medium).

### 2.5. Flow Cytometry Analysis

The cells at a density of 1.2 *×* 10^5^ cells/well were seeded in 6-well plates and incubated for 24 h. Afterward, the media of MDA-MB-231 and MCF-7 cells were replaced with fresh supplemented media containing 450 and 1000 µg/mL Mt, respectively. Following a 24 h incubation period, the cells were harvested and analyzed using Annexin V-FITC Apoptosis Detection and Cycletest™ Plus DNA Reagent Kits (BD Biosciences Pharmingen, San Diego, CA, USA).

### 2.6. qRT-PCR Analysis

The cells were seeded and exposed to Mt in the same way as for flow cytometry analysis, and experiments were conducted in three replicates from the same batch of cells. Total RNA isolation and cDNA synthesis were conducted using Trizol reagent (Roche, Munich, Germany) and High Capacity RNA-to-cDNA (Applied Biosystems, Waltham, MA, USA) Kits. qRT-PCR was performed with a Roche LightCycler 96 system using SYBR Select Master Mix (Applied Biosystems, Waltham, MA, USA), and the primers listed in Table S2.

### 2.7. Statistical Analysis

All experiments were conducted in three independent replicates. Data were statistically analyzed using Student’s *t*-test or ANOVA, followed by the Tukey post hoc test and shown as mean ± SD.

## 3. Results and Discussion

### 3.1. Properties of Mt

The Mt material used in the present study was exactly the same as used in our previous study [38], meaning that one Mt preparation was used in both of these studies. As shown in Figure S1, the Mt material was of good purity, composed of nanosheets with diameters of about 170–2100 nm, and exhibited a hydrodynamic size distribution of about 200–2250 nm with two population peaks centered at about 300 and 1000 nm. The Mt material with a CEC of 102 meq/100 g had a zeta potential of −40.1, −26.4, and −13.3 mV in a 0.1× phosphate buffered saline, RPMI and 10% FBS-RPMI, respectively.

### 3.2. Antiproliferative Effects

**Figure 1:**
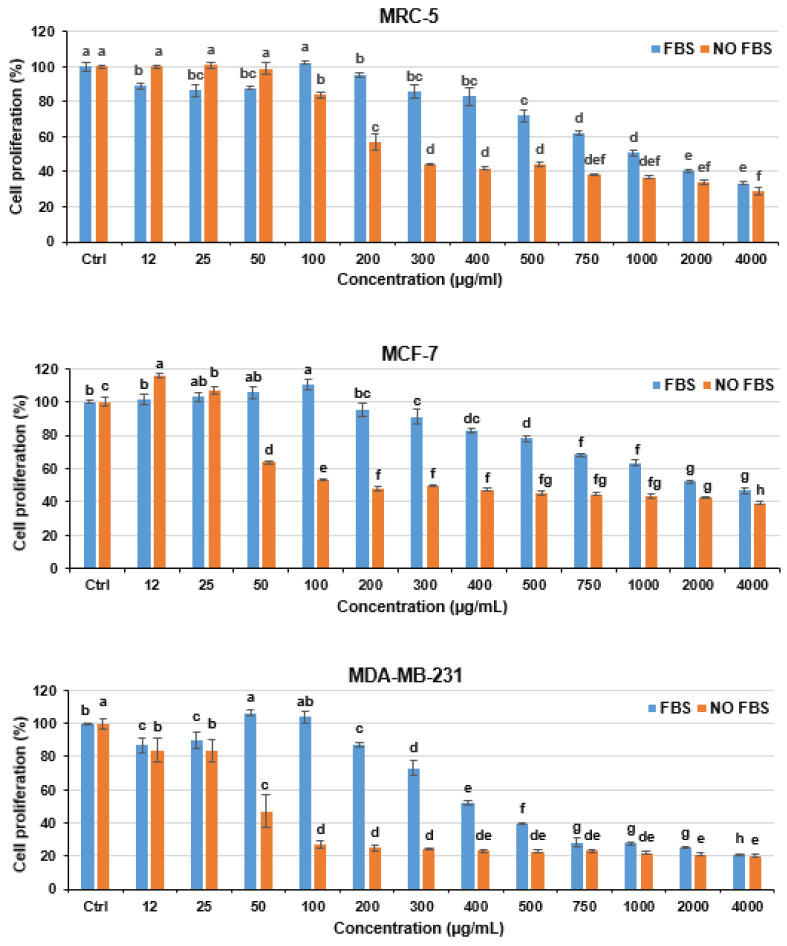
Antiproliferative effect of Mt on the cells in the media with and without 10% FBS following 24 h exposure. Data were statistically analyzed using the ANOVA methodology followed by the Tukey post hoc test and shown as mean ± SD. Bars with different superscripts are different at *p* ≤ 0.05, separately in each of the media.

The MTT assay results (Figure 1) showed that Mt at a wide range of concentrations inhibited the proliferation of MDA-MB-231 and MCF-7 cells to a degree, depending on its concentration, FBS content, and cell type. This antiproliferative effect was the same at 24 and 48 h (Figure S2). However, at some low concentrations, Mt slightly improved the development of MDA-MB-231 and MCF-7 cells. In the medium lacking FBS, Mt at 12–25 µg/mL improved the growth of MCF-7 cells; in the medium containing FBS, Mt at 50 and 100 µg/mL induced a growth in MDA-MB-231 and MCF-7 cells, respectively. This contradictory function of Mt was similarly observed in MRC-5, HepG2, and HT-29 cells [38] and is due to the abilities of Mt to (i) enter cells and induce cytotoxicity [40] and (ii) attach to the surface of a cell and provide growth signals [41]. These results disclose a dual concentration-dependent manner of Mt. This means that at the concentrations tolerable for cells and adequate for inducing growth signals, Mt causes cell growth, whereas it causes cell death at other concentrations.

As is evident from Figure 1, the function of Mt was affected by 10% FBS. In MCF-7 cells, FBS prevented the cell growth induced by Mt at concentrations of 12–25 µg/mL, and, in both cells, FBS dramatically decreased the antiproliferative activity of Mt at a broad range of concentrations. This phenomenon was also observed in MRC-5, HepG2, and HT-29 cells and is due to the affinity of Mt to adsorb proteins present in FBS, leading to reducing the contact between Mt nanosheets and cells and, consequently, decreasing the cell growth/death induced by Mt [38]. These results imply that the cytotoxicity of Mt can be different in areas with different protein levels in the body, such as the intestinal lumen, blood, and interstitial fluid. In support of this, Fröhlich et al. [42] showed that the cytotoxicity of carboxyl polystyrene nanoparticles was different in the media containing 0%, 1%, 2%, and 5% FBS: it decreased with the increase in FBS content due to the formation of protein corona on the nanoparticles.

Heat-inactivated FBS, used in the MTT assay, contains denatured bovine proteins that may interact with both the Mt and cell surfaces in a manner different from natural human proteins, which in turn may lead to different cytotoxicity of Mt. Hence, to evaluate cell sensitivity to Mt, the MTT assay data obtained from the FBS-free media may be more reliable. The MTT assay results of the present study and those of our previous study [38] show that the Mt IC_50_ for MDA-MB-231, HepG2, HT-29, MCF-7, and MRC-5 cells in the absence of FBS was about 50, 100, 150, 200, and 250 µg/mL, and in the presence of 10% FBS was about 400, 600, 850, 2000, and 1000 µg/mL, respectively. Therefore, among the above cell lines, the MDA-MB-231 cell line with a significant difference was the most vulnerable to Mt in both media, with and without FBS. In addition, the Mt concentrations inhibiting ~50% of MDA-MB-231 cells were non-toxic and very low toxic to normal lung fibroblast MRC-5 cells in the media with and without FBS, respectively. These results strongly suggest Mt as an antiproliferative nanoagent against MDA-MB-231 cells. Compared to Mt, fungus *Humicola*-based synthesized silver nanoparticles in concentrations of 500 and 1000 µg/mL suppressed about 10% and 40% of MDA-MB-231 cells [43], implying their low potency in inhibiting these cells. Some nanoparticles showed high toxicity to MDA-MB-231 cells; however, there were no data to show that these nanoparticles were less toxic to other cell types [1,11,12] or that these nanoparticles were as toxic or more toxic to other cell types. For instance, the nanoparticles developed by Syed et al. [43] in concentrations of 50–500 µg/mL were as toxic to normal embryonic fibroblast NIH3T3 cells as to MDA-MB-231 cells. Also, several nanoparticles that had high toxicity to MDA-MB-231 cells showed higher toxicity to U-87 (brain cancer), AGS (stomach cancer), and HFF (normal skin fibroblast) cells [44,45,46].

The most substantial difference between normal and cancer cells is their proliferation rate. The higher proliferation rate of cancer cells can be an ideal target to destroy them. Taking the results of our present and previous studies together, we found that there was a correlation between the proliferation rate of MDA-MB-231, HepG2, HT-29, MCF-7, and MRC-5 cells and the antiproliferative effect of Mt. This means that the cells with a higher proliferation rate were more vulnerable to Mt, which was more evident in the absence of FBS. The MDA-MB-231 cell line had the highest proliferation rate among the above cell lines, which might be the main reason for its highest vulnerability to Mt. In support of this, Fröhlich et al. [42], using 20 cell lines, showed that the cytotoxicity of carboxyl polystyrene nanoparticles was influenced by the properties of cells, including growth pattern, proliferation rate, size, and embryonic origin. They found that non-adherent cells and cells with a higher proliferation rate were significantly more vulnerable to the nanoparticles, while size and embryonic origin had less influence on their vulnerability. Nanoparticles may disrupt RNA transcription, protein synthesis, DNA replication, and cell division and, thus, further damage cells with higher proliferation rates [42,47]. In addition, cells with higher proliferation rates can be more sensitive to nanoparticle-induced mitochondrial damages, leading to ATP depletion, as discussed in the next section. Moreover, during cell division, internalized nanoparticles may be localized in one daughter cell rather than distributed in both daughter cells [48], which may increase the nanoparticle content to an intolerable level for the recipient cell. Also, a decrease in cell size following cell division may intensify vulnerability to nanoparticles [42].

Limitations of systemic cancer treatment led to the development of local cancer treatment strategies. Some benefits of local cancer treatment include improving the permeability and retention of therapeutic agents, reducing the administration frequency, reducing the systemic toxicity, and making it possible to treat inoperable tumors or cancers with high locoregional recurrence [49]. Direct injection of nanoclay-based anticancer agents into tumors and/or tumor resection sites is one of the emerging strategies for local cancer treatment [21,22,23,24,25]. Since Mt cytotoxicity is dependent on Mt concentration, protein level, and cell type, Mt may be an ideal candidate for local cancer treatment. When Mt is injected into a cancerous tissue, it can be lethal to cancer cells due to its high concentration, low protein level in interstitial fluid, high proliferation rate of cancer cells, and enhanced permeability and retention of Mt; however, when Mt gradually leaves the cancerous tissue and enters the circulatory system, it is expected that Mt is safe to the body due to its low concentration, high protein level in the blood, low proliferation rate of normal cells, and short residence time of Mt in the blood, as previous studies have shown no acute systemic toxicity of Mt even at oral and intravenous doses up to 5000 and 14.3 mg/kg, respectively [27,28,29]. Therefore, the injection of Mt-based anticancer products (e.g., pure Mt, drug-loaded Mt, Mt-based gels loaded with a drug, etc.) into inoperable tumors or tumor resection sites may be a feasible approach for the local or postoperative treatment of MDA-MB-231 breast cancer, as is supported by the promising results of in vivo studies on nanoclay-based anticancer products [21,22,23,24,25].

### 3.3. Cellular Mechanisms

**Figure 2:**
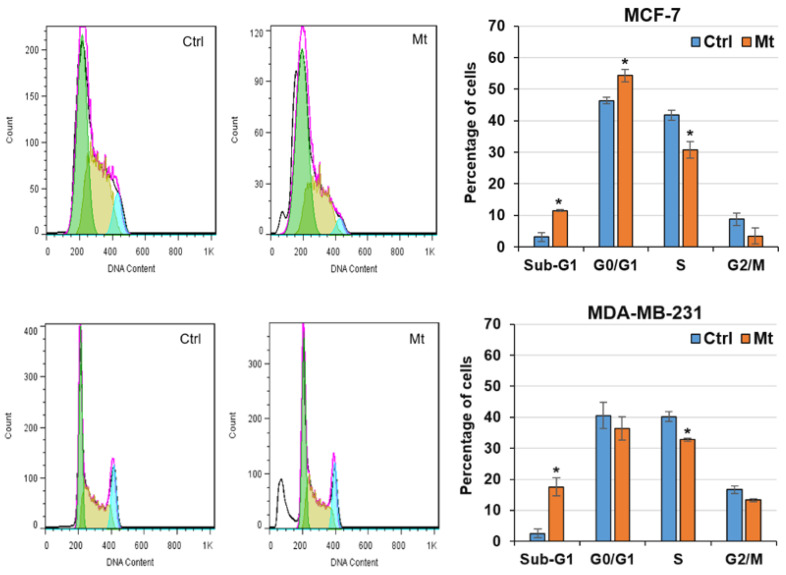
The effect of Mt on the cell cycle phase distributions, following exposure of the cells to Mt for 24 h in a 10% FBS medium. Representative flow cytometry histograms and graphed data, as mean ± SD of the three replicates, are displayed in the left and right panels. Significant differences at the level of * *p* ≤ 0.05 were determined by the Student’s *t*-test.

**Figure 3:**
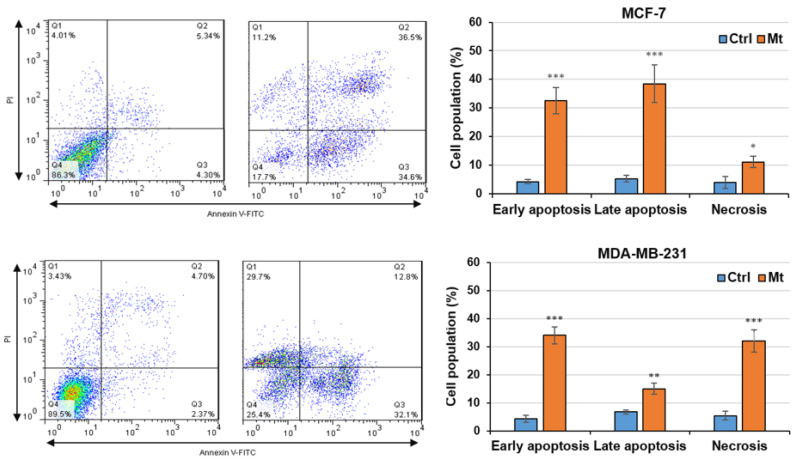
The effect of Mt on cell death, following exposure of the cells to Mt for 24 h in a 10% FBS medium. Representative flow cytometry dot plots and graphed data, as mean ± SD of the three replicates, are displayed in the left and right panels. Significant differences at the levels of * *p* ≤ 0.05, ** *p* ≤ 0.01, and *** *p* ≤ 0.001 were determined by the Student’s t-test.

Cell cycle and cell death in the untreated and Mt-treated cells were monitored by flow cytometry. The cell cycle data (Figure 2) show that Mt increased the sub-G1 populations from 2.5% and 3% to 17.5% and 11.5% in MDA-MB-321 and MCF-7 cells, respectively. Also, Mt increased the G0/G1 population from 46.4% to 54.3% in MCF-7 cells. The cell death data (Figure 3) show that Mt increased the apoptosis rates from 11% and 9.6% to 49% and 71% in MDA-MB-321 and MCF-7 cells, respectively. Also, Mt increased the necrosis rates from 5.4% and 4% to 32% and 11.2% in MDA-MB-321 and MCF-7 cells, respectively. Based on the flow cytometry findings, Mt induced sub-G1 arrest, apoptosis, and necrosis in both cells and G0/G1 arrest in MCF-7 cells. However, the necrosis and sub-G1 arrest rates were considerably higher in MDA-MB-231 cells, confirming the higher vulnerability of MDA-MB-231 cells to Mt.

ATP is the main energy source for the cell’s activities, including both cell growth and cell apoptosis [50]. It was shown that nanoparticle-induced oxidative stress can damage mitochondria [51] and that massive mitochondrial damage leading to failure of ATP generation can convert energy-dependent apoptosis to necrosis [50,51]. Since Mt can cause oxidative stress by generating reactive oxygen species (ROS) [52,53], the necrosis observed in the Mt-treated cells might be a result of ROS-induced mitochondrial damage leading to the failure of ATP generation. When mitochondria are damaged and ATP supply is limited, cells with higher proliferation rates and, thus, faster ATP depletion suffer from further necrosis. This may explain why the Mt-induced necrosis was higher in MDA-MB-231 cells (DT: 23.6 h) than in MCF-7 cells (DT: 42.1 h). Notably, with increasing the necrosis rate, the sub-G1 arrest also increased in the Mt-treated cells (Figure 2 and Figure 3). This provides further evidence of the possible damage to mitochondria that were responsible for generating ATP required for cell growth. In support of the latter, it was shown that both ROS- and parthenolide-induced mitochondrial damages led to sub-G1 arrest in B16-F10 and COLO205 cells, respectively [54,55]. Hence, mitochondrial damage leading to ATP depletion seems to be the most likely cause of the necrosis and sub-G1 arrest observed in the Mt-treated cells.

### 3.4. Molecular Mechanisms

**Figure 4:**
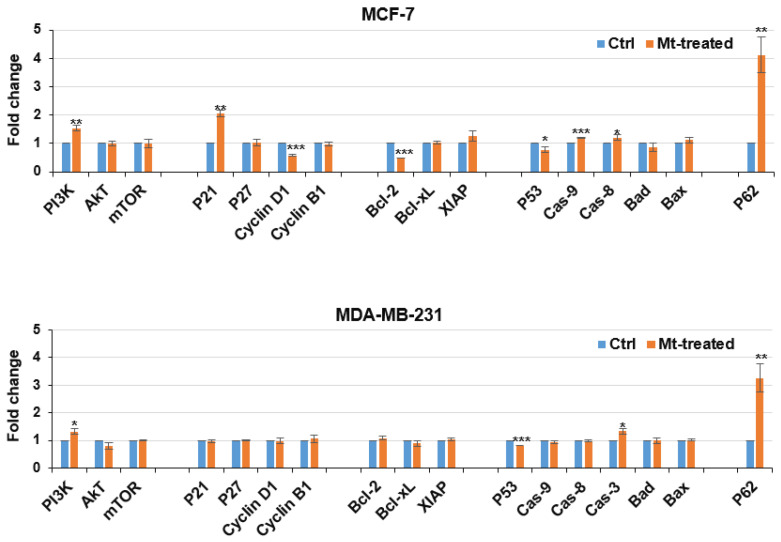
Relative gene expression levels in the cells untreated and treated with Mt for 24 h in a 10% FBS medium were obtained by qRT-PCR. Fold changes in Mt-treated cells relative to untreated cells were determined by 2^−ΔΔCt^ method after normalization to *HPRT1*. Values are given as mean ± SD of the three independent experiments. Significant differences at the levels of * *p* ≤ 0.05, ** *p* ≤ 0.01, and *** *p* ≤ 0.001 were determined by Student’s *t*-test.

The qRT-PCR results (Figure 4) show that Mt altered the expression of cell survival-, cell cycle-, and cell death-related genes in the cells. In MCF-7 cells, Mt up-regulated PI3K, *P21*, *Cas-9*, *Cas-8*, and *P62* by 1.5-, 2-, 1.2-, 1.2-, and 4.1-fold as well as down-regulated *Cyclin D1*, *Bcl-2*, and *P53* to 0.7-, 0.5-, and 0.8-fold, respectively. In MDA-MB-321 cells, Mt up-regulated *PI3K*, *Cas-3*, and *P62* by 1.3-, 1.3-, and 3.2-fold as well as down-regulated *P53* to 0.8-fold, respectively. These gene expression changes are in perfect accordance with the findings of the MTT assay (Figure 1) and flow cytometry (Figure 2 and Figure 3), as described in the following.

The signaling pathway of *PI3K*/*AKT*/*mTOR* converts extracellular stimulations into cellular functions involved in cell development and survival [56]. Therefore, the up-regulation of *PI3K* is in accordance with the growth induced by Mt in MCF-7 and MDA-MB-321 cells.

Cyclin D1 is required for the G0/G1 phase progression, and *P21* is involved in cell cycle arrest [57]. Therefore, the decreased *Cyclin D1* and increased *P21* expression justify the G0/G1 arrest in Mt-treated MCF-7 cells. A decrease in the *Bcl-2*/*Bax* ratio induces mitochondrial apoptosis [5,58], and apoptosis in MCF-7 cells lacking *Cas-3* expression can occur via the successive activation of *Cas-9*, *Cas-7*, and *Cas-6* [59]. Therefore, the decreased *Bcl-2* and increased *Cas-9* expression in Mt-treated MCF-7 cells imply the activation of the mitochondrial apoptosis pathway [5,58]. *P62* is an important protein in both the autophagy and autophagy-dependent apoptosis pathways [60,61]. There is evidence that inorganic nanoparticles can induce autophagy-dependent apoptosis mediated by *P62* [6]. Hence, the 4-fold increase in *P62* expression in the Mt-treated MCF-7 cells suggests the activation of the autophagy-dependent apoptosis pathway, in addition to the mitochondrial apoptosis pathway. In support of this, it was shown that the treatment of HCT 116 cells with selenium nanoparticles (SeNPs) led to the activation of both the mitochondria- and autophagy-dependent apoptosis pathways. As in the Mt-treated MCF-7 cells, the *Bcl-2*/*Bax* expression ratio was decreased, and the *P62* expression was elevated in the SeNPs-treated HCT 116 cells [62]. Taken together, the alterations in *Bcl-2*, *Cas-9*, and *P62* expression justify the apoptosis observed in Mt-treated MCF-7 cells.

After treatment with Mt, fewer gene expression changes were observed in MDA-MB-231 cells, which is in accordance with the higher Mt-induced necrosis observed in MDA-MB-231 cells. *P53* is involved in cell survival [1], *P62* can act as an apoptosis inducer [57], and *Cas-3* is involved in diverse apoptosis pathways [5]. Therefore, the decreased *P53* and increased *P62* and *Cas-3* expression justify the apoptosis observed in Mt-treated MDA-MB-231 cells.

## 4. Conclusions

The potential of Mt as an antiproliferative nanoagent against MDA-MB-231 and MCF-7 human breast cancer cells was investigated here. Although both MDA-MB-231 and MCF-7 are cell lines of breast cancer, Mt inhibited the proliferation of MDA-MB-231 and MCF-7 cells differently. Mt induced higher rates of sub-G1 arrest and necrosis in MDA-MB-231 cells, and MDA-MB-231 cells were much more vulnerable to Mt. In addition, MDA-MB-231 cells appeared more vulnerable to Mt in comparison with MRC-5, HepG2, and HT-29 cells. The higher vulnerability of MDA-MB-231 cells to Mt was probably due to their higher proliferation rate, which is the main distinguishing feature of cancer cells that can be targeted to destroy them. In addition, Mt cytotoxicity was highly dependent on the Mt concentration and serum level, along with the cell type, thus making Mt an ideal candidate for local treatment of MDA-MB-231 cells. These findings show the potential of biocompatible Mt to kill highly aggressive MDA-MB-231 cells, which may be useful in the development of local nanoparticle-based therapies.

## Data Availability

The datasets generated during and/or analyzed during the current study are available from the corresponding author on reasonable request.

## Supplementary Materials

The following supporting information can be downloaded at https://www.mdpi.com/article/10.3390/cells13020200/s1, Figure S1 Properties of Mt. (**a**) XRD patterns of bentonite, extracted Mt and purified Mt, from bottom to top. The reflection of quartz (Q) disappeared in extracted Mt, indicating the high efficiency of the extraction method. The (001) reflection of Mt was weakened and shifted towards higher angle after purification, implying the partial exfoliation of Mt nanosheets and the removal of interlayer impurities; (**b**) Hydrodynamic size distribution based on dynamic light scattering (DLS) measurement; (**c**) FESEM image of separated Mt nanosheets. Although DLS data estimate the size of spherical particles, the size distribution determined by DLS is in accordance with the FESEM data. Figure S2: Antiproliferative effect of Mt on the cells in media containing 10% FBS after 24 and 48 h. Data were statistically analyzed by ANOVA methodology followed by Tukey post-hoc test and shown as mean ± SD. No significant differences were observed between 24 and 48 h exposure times (*p* ≤ 0.05). Table S1 Chemical composition of bentonite. Table S2 Primers used for qRT-PCR analysis.
