# Supplementary material for: Exploring the Potential of Montmorillonite as an Antiproliferative Nanoagent against MDA-MB-231 and MCF-7 Human Breast Cancer Cells
Source: Cells. 2024 Jan 22;13(2):200. doi: 10.3390/cells13020200 (PMC10814472; doi:10.3390/cells13020200)
Supplement: Supplementary file 1 [file cells-13-00200-s001.zip › cells-2754122-supplementary.pdf]

Supplementary Information

Table S1 Chemical composition of bentonite

Formula	Wt.%	Formula	Wt.%
SiO_2	61.03	SO_2	0.37
Al_2O_3	14.59	Cl	0.46
Fe_2O_3	2.09	K_2O	0.76
Cao	0.77	TiO_2	0.22
MgO	2.22	BaO	0.11
Na_2O	2.04	Loss of ignition	13.2

Table S2 Primers used for qRT-PCR analysis

Gene	Primer sequences (5'-3')
AkT	F: TCTATGGCGCTGAGATTGTG<br>R: CTTAATGTGCCCGTCCTTGT
PI3K	F: CCTGATCTTCCTCGTGCTGCTC<br>R: ATGCCAATGGACAGTGTTCCTCTT
mTOR	F: CTGGGACTCAAATGTGTGCAGTTC<br>R: GAACAATAGGGTGAATGATCCGGG
P21	F: *TACCCTTGTGCCTCGCTCAG*<br>R: *GAGAAGATCAGCCGGCGTTT*
P27	F: AGCGACCTGCTGCAGAAGAT<br>R: TTACGTCTGGCGTCGAAGGC
Cyclin D1	F: TCCGGCGAGGGGCAGAAGAG<br>R: GCAGCACCCGGTCGTTGAGGA
Cyclin B1	F: AAAGGCGTAACTCGAATGGA<br>R: CCGACCTTTTATTGAAGAGCA
Bcl-2	F: CTGCACCTGACGCCCTTCACC<br>R: CACATGACCCCACCGAACTCAAAG
Bcl-xL	F: CCCAGAAAGGATACAGCTGG<br>R: GCGATCCGACTCACCAATAC
XIAP	F: ATAGTGCCACGCAGTCTACAA<br>R: AGATGGCCTGTCTAAGGCAAA
P53	F: GAGGTTGGCTCTGACTGTACC<br>R: TCCGTCCCAGTAGATTACCAC
Cas-9	F: ATGGACGAAGCGGATCGG<br>R: CCCTGGCCTTATGATGTT
Cas-8	F: CATTTGCATATTTAGCCGCCAAG<br>R: TTAAGAGTCCCAGGAATTCAGCAAC
Cas-3	F: GACTCTGGAATATCCCTGGACAACA<br>R: AGGTTTGCTGCATCGACATCTG
Bad	F: CCCAGAGTTTGAGCCGAGTG<br>R: CCCATCCCTTCGTCGTCCT
Bax	F: CCGGCGAATTGGAGATGAAC<br>R: CCCCAGTTGAAGTTGCCATC
P62	F: AGGCGCACTACCGCGAT<br>R: CGTCACTGGAAAAGGCAACC
HPRT1	F: GCTATAAATTCTTTGCTGACCTGCTG<br>R: AATTACTTTTATGTCCCCTGTTGACTGG

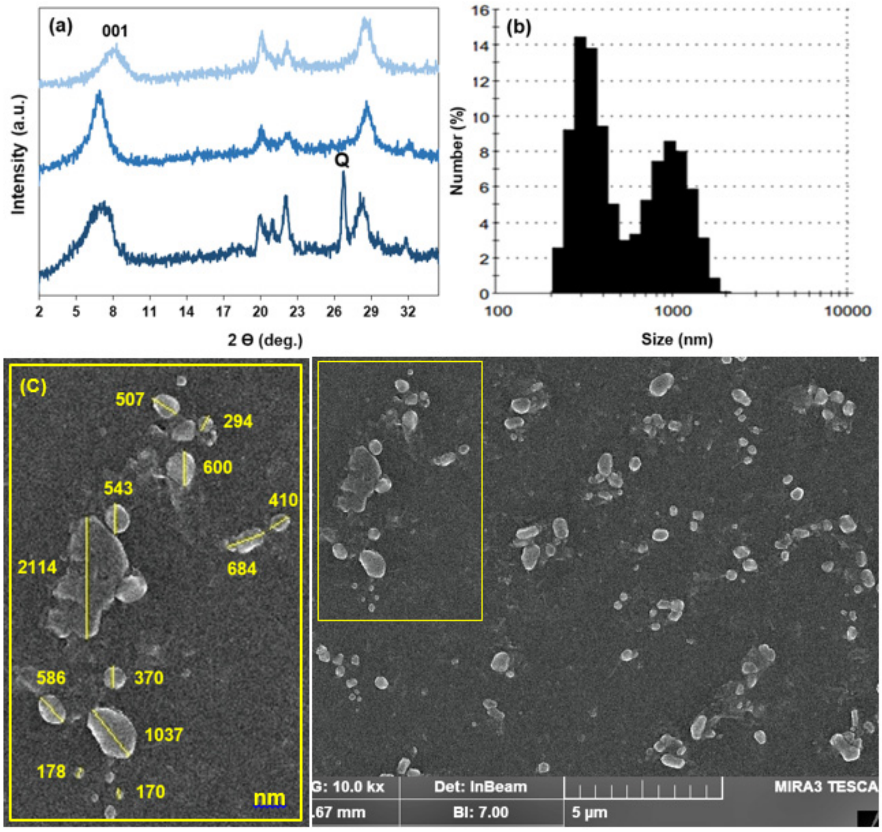

Fig. S1 Properties of Mt. (a) XRD patterns of bentonite, extracted Mt and purified Mt, from bottom to top. The reflection of quartz (Q) disappeared in extracted Mt, indicating the high efficiency of the extraction method. The (001) reflection of Mt was weakened and shifted towards higher angle after purification, implying the partial exfoliation of Mt nanosheets and the removal of interlayer impurities; (b) Hydrodynamic size distribution based on dynamic light scattering (DLS) measurement; (c) FESEM image of separated Mt nanosheets. Although DLS data estimate the size of spherical particles, the size distribution determined by DLS is in accordance with the FESEM data

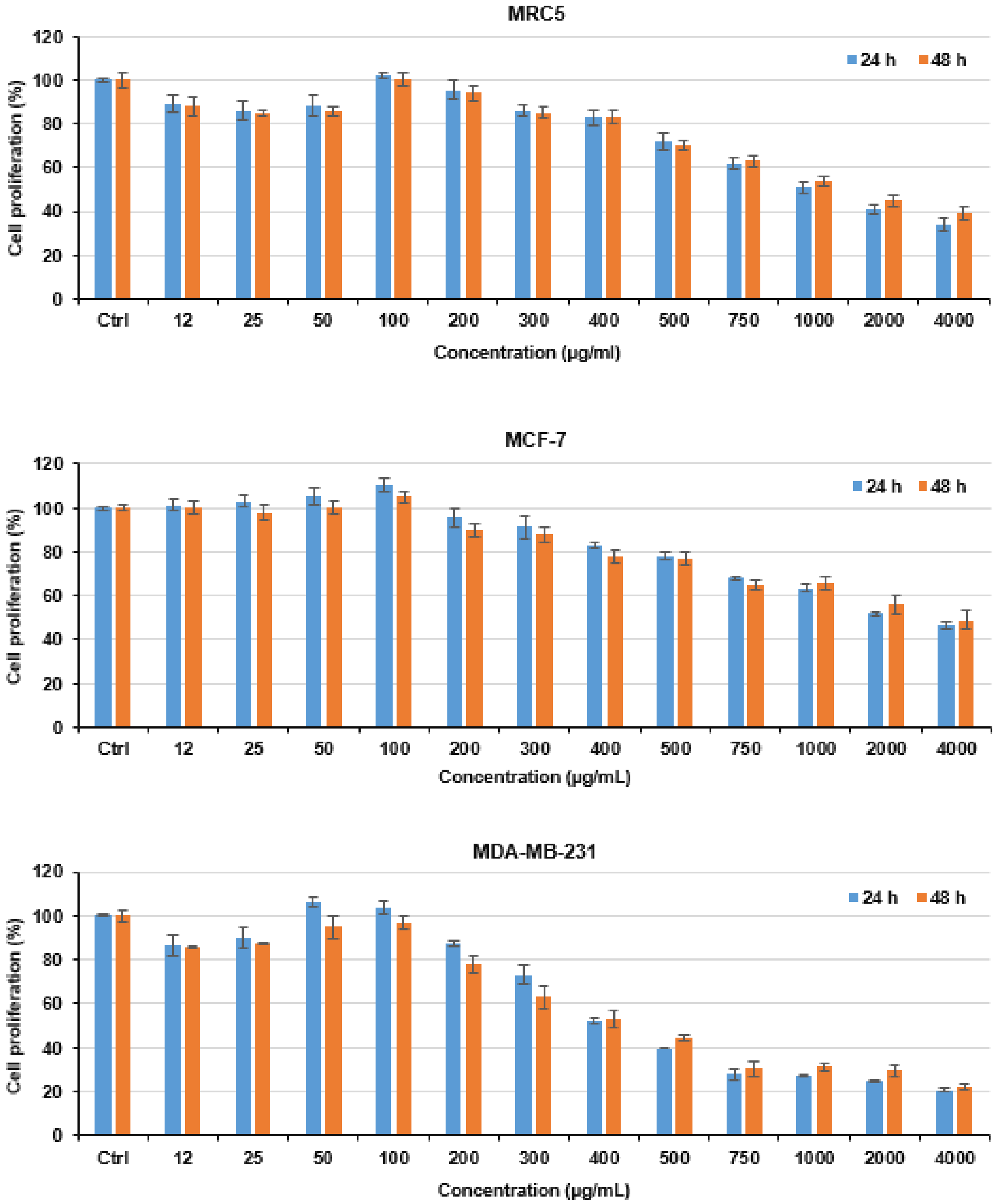

Fig. S2 Antiproliferative effect of Mt on the cells in media containing 10% FBS after 24 and 48 h. Data were statistically analyzed by ANOVA methodology followed by Tukey post-hoc test and shown as mean ± SD. No significant differences were observed between 24 and 48 h exposure times ($p \leq 0.05$).
